# Supplementary material for: Optimization of differentiation and transcriptomic profile of THP-1 cells into macrophage by PMA
Source: PLoS One. 2023 Jul 17;18(7):e0286056. doi: 10.1371/journal.pone.0286056 (PMC10351730; doi:10.1371/journal.pone.0286056)
Supplement: S1 File — (ZIP) [file pone.0286056.s001.zip › supporting files/RNA-seq report.zip!/RNA-seq report/4_GOEnrichment_result/induce_vs_control/DAG/induce_vs_control.DEG_down_cc_DAG.pdf]

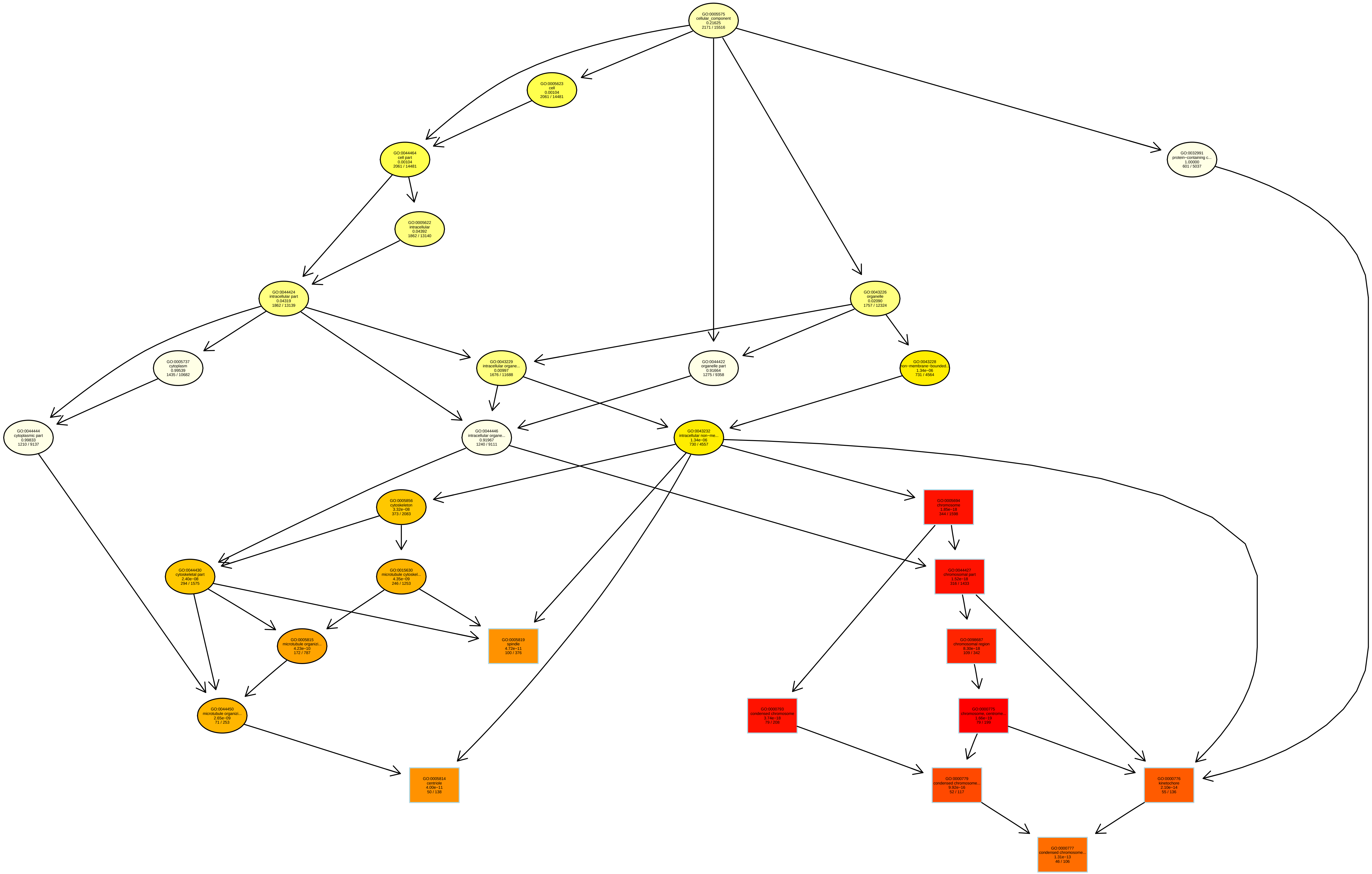
GO:0005575
cellular_component
0.21625
2171 / 15516
GO:0005623
cell
0.00104
2061 / 14481
GO:0044464
cell part
0.00104
2061 / 14481
GO:0032991
protein−containing c...
1.00000
601 / 5037
GO:0005622
intracellular
0.04392
1862 / 13140
GO:0044424
intracellular part
0.04319
1862 / 13139
GO:0043226
organelle
0.02090
1757 / 12324
GO:0005737
cytoplasm
0.99539
1435 / 10682
GO:0043229
intracellular organe...
0.00997
1676 / 11688
GO:0044422
organelle part
0.91664
1275 / 9358
GO:0043228
non−membrane−bounded...
1.34e−06
731 / 4564
GO:0044444
cytoplasmic part
0.99833
1210 / 9137
GO:0044446
intracellular organe...
0.91967
1240 / 9111
GO:0043232
intracellular non−me...
1.34e−06
730 / 4557
GO:0005856
cytoskeleton
3.32e−08
373 / 2083
GO:0005694
chromosome
1.85e−18
344 / 1598
GO:0044430
cytoskeletal part
2.40e−08
294 / 1575
GO:0015630
microtubule cytoskel...
4.35e−09
246 / 1253
GO:0044427
chromosomal part
1.52e−18
316 / 1433
GO:0005815
microtubule organizi...
4.23e−10
172 / 787
GO:0005819
spindle
4.72e−11
100 / 376
GO:0098687
chromosomal region
8.30e−18
109 / 342
GO:0044450
microtubule organizi...
2.65e−09
71 / 253
GO:0000793
condensed chromosome
3.74e−18
79 / 208
GO:0000775
chromosome, centrome...
1.66e−19
79 / 199
GO:0005814
centriole
4.00e−11
50 / 138
GO:0000779
condensed chromosome...
9.92e−16
52 / 117
GO:0000776
kinetochore
2.10e−14
55 / 136
GO:0000777
condensed chromosome...
1.31e−13
46 / 106
